# Histamine 2/3 receptor agonists alleviate perioperative neurocognitive disorders by inhibiting microglia activation through the PI3K/AKT/FoxO1 pathway in aged rats

**DOI:** 10.1186/s12974-020-01886-2

**Published:** 2020-07-22

**Authors:** Yi-Nan Chen, Huan-Huan Sha, Yi-Wei Wang, Qin Zhou, Piplu Bhuiyan, Na-Na Li, Yan-Ning Qian, Hong-Quan Dong

**Affiliations:** 1 Department of Anesthesiology, The First Affiliated Hospital of Nanjing Medical University, Nanjing, 210029 Jiangsu People’s Republic of China; 2 Department of Anesthesiology, Wuxi People’s Hospital, Wuxi, 214001 Jiangsu People’s Republic of China; 3 Department of Anesthesiology, Jiangsu Cancer Hospital, Nanjing, 210009 Jiangsu People’s Republic of China

**Keywords:** Microglia, Histamine receptors, Perioperative neurocognitive disorders, Inflammatory factors, TLR4, FoxO1

## Abstract

**Background:**

Microglia, the principal sentinel immune cells of the central nervous system (CNS), play an extensively vital role in neuroinflammation and perioperative neurocognitive disorders (PND). Histamine, a potent mediator of inflammation, can both promote and prevent microglia-related neuroinflammation by activating different histamine receptors. Rat microglia express four histamine receptors (H1R, H2R, H3R, and H4R), among which the histamine 1 and 4 receptors can promote microglia activation, whereas the role and cellular mechanism of the histamine 2 and 3 receptors have not been elucidated. Therefore, we evaluated the effects and potential cellular mechanisms of histamine 2/3 receptors in microglia-mediated inflammation and PND.

**Methods:**

This study investigated the role of histamine 2/3 receptors in microglia-induced inflammation and PND both in vivo and in vitro. In the in vivo experiments, rats were injected with histamine 2/3 receptor agonists in the right lateral ventricle and were then subjected to exploratory laparotomy. In the in vitro experiments, primary microglia were pretreated with histamine 2/3 receptor agonists before stimulation with lipopolysaccharide (LPS). Cognitive function, microglia activation, proinflammatory cytokine production, NF-κb expression, M1/M2 phenotypes, cell migration, and Toll-like receptor-4 (TLR4) expression were assessed.

**Results:**

In our study, the histamine 2/3 receptor agonists inhibited exploratory laparotomy- or LPS-induced cognitive decline, microglia activation, proinflammatory cytokine production, NF-κb expression, M1/M2 phenotype transformation, cell migration, and TLR4 expression through the PI3K/AKT/FoxO1 pathway.

**Conclusion:**

Based on our findings, we conclude that histamine 2/3 receptors ameliorate PND by inhibiting microglia activation through the PI3K/AKT/FoxO1 pathway. Our results highlight histamine 2/3 receptors as potential therapeutic targets to treat neurological conditions associated with PND.

## Introduction

Perioperative neurocognitive disorders (PND), common complications that occur after surgery, have recently received significant attention. Patients over the age of 65 who underwent noncardiac surgery had a 26% prevalence of PND within a few weeks, which decreased to 10% 3 months after surgery [1]. PND are characterized by impaired concentration, learning, and memory and are also associated with various adverse outcomes, including prolonged hospitalization, increased complications and mortality, and decreased quality of life [2]. Neuroinflammation has been identified as an important process associated with the occurrence and development of PND [3].

Microglia, the principal resident immune cells of the central nervous system (CNS), are likely to play a vital role in promoting CNS pathology [4–7]. Different types of stimuli can activate microglia, which further promote the acceleration of neuroinflammation and exacerbation of brain disorders [8, 9]. In different microenvironments, microglia exhibit different M1/M2 phenotypes [10], and an efficient balance between these phenotypes is necessary to restore CNS homeostasis [11–13]. Hence, inhibiting microglia overactivation and maintaining the balance of microglia phenotypes may provide a novel therapeutic strategy to improve the treatment of PND.

Histamine is an endogenous biogenic amine that acts as an important inflammatory mediator in the brain [14–16]. In the brain, histamine can be produced by histaminergic neurons, mast cells, and microglia and plays dual roles in regulating microglia-mediated inflammatory responses [17–19]. Histamine exerts its effects by activating four distinct subtypes of G protein-coupled receptors via interacting with specific receptors (H1R, H2R, H3R, and H4R) that are differentially expressed by microglia [20, 21]. Dong et al. showed that H1R and H4R stimulated microglia activation and subsequent production of TNF-α and IL-6 through the MAPK and PI3K/AKT–NF-κb pathways [22]. However, the mechanisms of H2R and H3R in microglia-mediated inflammation remain unclear.

TLR4 is a member of the Toll-like receptor (TLR) family and is widely expressed in the CNS, including in microglia, neurons, astrocytes, and endothelial cells [23, 24]. As a pathogen-associated molecular pattern-recognizing receptor, TLR4 can identify pathogens and endogenous molecules, such as lipopolysaccharide (LPS) and cytokines, thereby inducing the expression of downstream signaling proteins and leading to the massive release of inflammatory cytokines. An increasing number of studies have shown a dominant role of TLR4 in activating microglia and inducing inflammation [25–27]. FoxO1, also known as FKHR, along with FoxO3 and FoxO4, constitutes the O class of the forkhead transcription factor family (FoxO). FoxO1 is downstream of TLR4-mediated signaling. Activation of TLR4-PI3K/Akt can phosphorylate FoxO1, induce the nuclear exclusion of FoxO1, and further suppress the expression of TLR4; this process is called the “self-limiting mechanism” of the TLR4-PI3K/Akt-FoxO1 axis [28–31]. FoxO1-induced inhibition of TLR4 can inactivate downstream proinflammatory signaling pathways and protect the stability of the CNS.

The role of histamine in microglia-induced inflammation has been widely studied in recent years. However, the effects and mechanisms of histamine receptors in inflammation have not been systematically studied. In the present study, we investigated the functional role of H2R/H3R in microglia-mediated inflammation both in vivo and in vitro. Moreover, we suggested a possible signaling pathway involved in the anti-inflammatory effects associated with H2R/H3R.

## Materials and methods

### Animals

Forty-eight aged male Sprague-Dawley (SD) rats (12 months old, each weighing approximately 300–400 g) were used in this study and were obtained from the Model Animal Research Center of Nanjing University. All animals were housed in groups of five animals per cage under standard laboratory conditions, including free access to food and water, constant room temperature of 22 °C, humidity of 50–60%, and a 12:12-day–night cycle. All experiments were performed according to the Guide for the Care and Use of Laboratory Animals of the National Institutes of Health and the Guidelines for the Care and Use of Animals in Neuroscience Research by the Society for Neuroscience. The experiments were approved by the IACUC (Institutional Animal Care and Use Committee of Nanjing Medical University, No. 1909013).

### Reagents

Dulbecco’s modified Eagle’s medium (DMEM) and fetal calf serum (FCS) were purchased from Gibco–BRL (Grand Island, NY, USA). Lipopolysaccharide (LPS) was purchased from Sigma-Aldrich (St. Louis, MO, USA), and the H2R agonist amthamine dihydrobromide (amthamine) and the H3R agonist (R)-(−)-α-methylhistamine dihydrobromide ((R)-(−)-α-methylhistamine) were purchased from Tocris Bioscience (Bristol, UK). Fluoroshield mounting medium with DAPI was purchased from Abcam (HK, China). The transwell migration assays were purchased from Corning, Inc. (Lowell, MA, USA). The rat IL-1β, TNF-α, and IL-10 immunoassay kits were obtained from R&D Systems, Inc. (Minneapolis, MN, USA). The PI3K/AKT inhibitor wortmannin was purchased from R&D Systems, Inc. (Minneapolis, MN, USA). Adv-null and Adv-FoxO1 were purchased from GenePharma (Shanghai, China). The rabbit anti-Iba1 antibody was purchased from Wako, Ltd. (Osaka, Japan). Specific rabbit anti-H2 receptor antibodies were purchased from Alomone Labs Ltd. (Israel), and specific rabbit anti-H3 receptor antibodies were purchased from Abcam (Hong Kong, China). Specific rabbit antibodies against TLR4, FoxO1, NF-κB-p65, histone, and β-actin were obtained from Cell Signaling (Beverly, MA, USA). The goat anti-mouse secondary antibody and the goat anti-rabbit secondary antibody were obtained from Yfxbio (NJ, China). Goat anti-rabbit Alexa Fluor 594 secondary antibodies were purchased from Invitrogen (Carlsbad, CA, USA).

### In vivo experiments

#### Surgery and drug administration

Forty-eight male rats were randomly assigned to four groups (groups A–D) with 12 rats in each group. This study was performed in a double-blind manner. The rats were anesthetized with 50 mg/kg pentobarbital sodium that was administered intraperitoneally and then placed in a stereotaxic apparatus (Stoelting Instruments, United States). Guide cannulas (Plastic One) were inserted into the right lateral ventricle of the rats at 0.80 mm rostral and 1.50 mm lateral to the bregma and at a depth of 3.70 mm from the dorsal surface of the skull. After implantation, the rats were given 7 days to recover, with daily handling to evaluate the guide cannula.

For the experiments, drugs were injected directly into the right lateral ventricle through the implanted guide cannulas. The rats in groups A–B were pretreated with a site-directed injection of saline (5 μl), while the rats in group C were pretreated with the histamine 2 receptor agonist amthamine dihydrobromide (10 mM, 5 μl), and the rats in group D were pretreated with the histamine 3 receptor agonist (R)-(−)-α-methylhistamine dihydrobromide (10 mM, 5 μl). The rats were kept in their cages for 30 min without additional restraint. Then, the rats in groups B, C, and D were subjected to exploratory laparotomy. One day after drug administration, the rats were sacrificed, and the brains were collected for morphological and biochemical analyses.

Exploratory laparotomy was performed after the rats were anesthetized with 50 mg/kg pentobarbital sodium, which was administered intraperitoneally. Briefly, the abdomen of each rat was shaved, and a 3-cm midline incision was made to penetrate the peritoneal cavity. The wound was vigorously dilated by inserting a 5-cm sterile cotton swab into the peritoneal cavity. Approximately, 10 cm of the small intestine was exteriorized and left in the air for 20 min. The intestine was then placed inside the peritoneal cavity, and the wound was sutured in three layers consisting of the peritoneal lining, abdominal muscles, and skin. Exterior wounds were dressed with antibiotic powder. Sham-treated animals were only anesthetized and shaved.

#### Immunofluorescence analysis

The rats were anesthetized with pentobarbital sodium and then perfused with 0.9% NaCl, followed by cold 4% paraformaldehyde in 0.1 M phosphate-buffered saline (PBS) at pH 7.4. The brains were removed and stored overnight in 4% paraformaldehyde and then cryopreserved in PBS containing 30% sucrose before being stored at − 70 °C. Before acquiring images, the brains were trimmed into small blocks with thicknesses of less than 4 mm. Then, the brain sections were processed for immunofluorescence. After incubation for 1 h in 10% bovine serum albumin with 0.3% Triton X-100 in 0.01 M PBS, the brain sections were incubated overnight with rabbit anti-Iba1 and anti-TLR4 antibodies (1:200) in blocking solution at 4 °C. The tissue sections were washed three times with PBS and then incubated with goat anti-rabbit secondary antibody (1:1000) at 37 °C for 1 h. The cell nuclei were stained with DAPI. Fluorescent images were acquired using a confocal microscope. The numbers of Iba1-positive cells were determined with the Cell D software (Olympus) and are expressed as the number of cells in the high-power field.

#### Trace fear conditioning (TFC)

Forty-eight rats were trained to associate an unconditioned stimulus (foot shock) and a conditioned stimulus (tone) with the environment. H2R/H3R agonists were administered immediately after fear conditioning, and exploratory laparotomy was performed 30 min later. The training consisted of placing the rat in the conditioning chamber and allowing the rat to explore the surroundings for 100 s. Next, the conditioned stimulus, an auditory cue (80 dB, 5 kHz), was presented for 20 s. The unconditioned stimulus, a 2-s foot shock (0.8 mA), was administered after termination of the tone. This procedure was repeated with an interval of 100 s, and the rats were removed from the chamber 100 s later. Rats anticipate the shock by “freezing”, which is defined as the absence of all movement except respiration; this defensive posture reflects learned fear. When placed in the same context on a subsequent occasion, the learned fear is recalled, and the degree of learning and recall can be determined based on the extent of freezing. Contextual memory of the learned fear was assessed 1 day after the exploratory laparotomy, and freezing behavior in the absence of the tone and shock was automatically scored by the video tracking software (Xeye Fcs, Beijing MacroAmbition S&T Development Co., Ltd., Beijing, China) over the course of 300 s. The freezing scores for each subject are expressed as a percentage of the total testing time.

### In vitro experiments

#### Preparation of microglia-enriched cultures

Briefly, tissues from whole brains of postnatal (P1-P2) Sprague-Dawley rats were triturated, and the cells were plated in poly-d-lysine-precoated cell culture flasks in high-glucose Dulbecco’s modified Eagle’s medium (DMEM) containing 10% fetal calf serum, 100 U/ml penicillin, and 100 mg/ml streptomycin. Cultures were maintained at 37 °C in a humidified atmosphere of 5% CO_2_ and 95% air. After forming a confluent monolayer of glial cells (10–14 days), the microglia were separated from the astrocytes by shaking for 5 h at 100 rpm and were replated in 24-well culture plates at a density of 10^5^ cells/cm^2^. The purity of the microglia was > 98%, as confirmed by anti-Iba1 immunochemical staining.

#### Adenovirus transduction

Primary microglia were transduced with adenoviral vectors at a defined multiplicity of infection (MOI). The adenoviral vectors used were as follows: Adv-CMV-FoxO1 expressing wild-type FoxO1 (1.0 × 10^11^ pfu/ml) and the null adenovirus Adv-null (1.25 × 10^11^ pfu/ml). After 48 h of incubation, the cells were collected for analysis.

#### Microglia challenge

Microglia were incubated with serum-free basal medium for 6 h before challenge. For the challenge experiments, the microglia were exposed to LPS (10 ng/ml) for 24 h. For certain experiments, the microglia were preincubated with the H2R agonist amthamine or H3R agonist (R)-(−)-α- methylhistamine (10 μM) for 30 min before the addition of LPS. After 24 h of LPS incubation, the culture supernatants were collected for ELISA analysis, q-PCR, immunofluorescence experiments, and migration experiments. For the cell signaling experiments, the cultured microglia were pretreated with 10 μM H2R agonist or H3R agonist 30 min before the addition of LPS (10 ng/ml) and were then collected for analysis 24 h later. Alternatively, the microglia were pretreated with the PI3K inhibitor wortmannin for 30 min or adenovirus for 48 h before exposure to the H2R/H3R agonists and LPS.

#### TNF-ɑ, IL-1β and IL-10 assays

The amounts of TNF-α, IL-1β, and IL-10 were measured with commercial ELISA kits from R&D Systems. All reagents and samples were brought to room temperature before testing. Assay diluent (50 μL) was added to each well, and then standards, controls, or samples (50 μL) were added to each well. After incubation for 2 h at room temperature, each well was washed five times with wash buffer (400 μL each time). After the last wash, the remaining wash buffer was removed by inverting the plate and blotting it against clean paper towels. Rat TNF-α/IL-1β/IL-10 conjugate (100 μL) was added to each well. After incubation for 2 h at room temperature, each well was washed. Substrate solution (100 μL) was added to each well and incubated for 30 min at room temperature without light. Stop solution (100 μL) was added to each well. The optical density of each well was measured within 30 min using a microplate reader set to 450 nm.

#### Western blotting

Hippocampal tissues and microglia were homogenized in RIPA lysis buffer, which contained 50 mM Tris, 150 mM NaCl, 1% Triton X-100, 2 mM EDTA, 1.5 μg/mL leupeptin, and 1 mM phenylmethylsulfonyl fluoride. The lysate was centrifuged for 20 min at 12,000×*g* and 4 °C. The protein level was determined by a BCA assay (Thermo Scientific, Waltham, MA, USA), and 20 μg of protein was loaded in each lane of a modified gel for analysis by sodium dodecyl sulfate polyacrylamide gel electrophoresis. The separated proteins were transferred onto polyvinylidene difluoride membranes (Millipore, Bedford, MA, USA), which were then blocked for 1 h with 5% nonfat milk in tris-buffered saline with Tween 20. The blocked membranes were probed overnight with specific primary antibodies diluted in 5% nonfat milk according to the manufacturer’s recommendation. The primary antibodies used were rabbit anti-H2 receptor (1:200), rabbit anti-H3 receptor (1:1,000), rabbit anti-FoxO1 (1:1,000), rabbit anti-NF-κB-p65 (1:1,000), rabbit anti-TLR4 (1:1,000), rabbit anti-histone (1:1,000), and rabbit anti-β-actin (1:1,000). The membranes were washed and incubated with secondary antibodies (1:1,000) and were also washed and incubated with ECL reagent before being exposed to film. Densitometry analysis was performed with the Image Lab software (Bio-Rad, Richmond, CA, USA) and quantified using the gel analysis plugin for ImageJ (NIH, Bethesda, MD, USA).

#### Q-PCR

Total RNA was extracted from primary microglia using TRIzol reagent (Invitrogen), and reverse transcription was performed with 1 μg of total RNA for each sample using the Transcription First Strand cDNA Synthesis Kit (Roche) according to the manufacturer’s instructions. q-PCR amplification was performed using a StepOne Plus Real-Time PCR System (Applied Biosystems) with SYBR Green master mix (Applied Biosystems, Foster City, CA) in a final volume of 10 μl that contained 1 μl of cDNA template from each sample. The primers used were as follows: rat β-actin forward, 5′-CCCATCTATGAGGGTTACGC-3′ and reverse, 5′-TTTAATGTCACGCACGATTTC-3′; rat TNF-α forward, 5′-CATCCGTTCTCTACCCAGCC-3′ and reverse, 5′-AATTCTGAGCCCGGAGTTGG-3′; rat IL-1β forward, 5′-GACTTCACCATGGAACCCGT-3′ and reverse, 5′-GGAGACTGCCCATTCTCGAC-3′; rat iNOS forward, 5′-TCCTCAGGCTTGGGTCTTGT-3′ and reverse, 5′-AGAAACTTCCAGGGGCAAGC-3′; and rat CD206 forward, 5′-TGTGAGCAACCACTGGGTTA-3′ and reverse, 5′-GTGCATGTTTGGTTTGCATC-3′. The cycling conditions were 95 °C for 10 min, followed by 40 cycles of 95 °C for 15 s and 60 °C for 1 min. The relative mRNA values were normalized to those of the control gene β-actin and calculated using the comparative cycle threshold (ΔΔCt) method.

#### Immunofluorescence

To determine the effect of LPS and H2R/H3R receptor agonists on microglia activation, primary microglia were fixed with 4% paraformaldehyde for 30 min. Nonspecific binding was blocked by incubating the cells in 5% BSA and 0.1% Triton X-100 solution for 1 h at room temperature. Then, the cells were incubated with anti-Iba1 (1:300) overnight at 4 °C. After three washes with PBS, the microglia were incubated with goat anti-rabbit Alexa Fluor 594 secondary antibody (1:1000), and the nuclei were stained with DAPI. Fluorescent images were acquired by using a confocal microscope.

#### Transwell migration assay

Transwell migration assays were performed using inserts with 8-μm diameter pores (Corning, Lowell, MA). Briefly, 2 × 10^5^ microglia cells were plated in the upper chamber in 200 μl of serum-free medium. This upper chamber was then placed in the bottom wells, which contained 600 μl of medium containing LPS. Then, H2R/H3R receptor agonists (10 μM) were added to the medium of the upper chamber. After incubation at 37 °C for 24 h, the nonmigrating cells on the upper surface of the membrane were carefully removed with a cotton swab. The cells on the lower surface of the membrane were first fixed in 4% paraformaldehyde for 30 min and then stained with 0.2% crystal violet for 1 h. For quantification, three randomly selected fields on the lower surface of the membrane were imaged using computer-assisted microscopy.

### Statistical analysis

The data are expressed as the mean ± SEM. Statistical analysis was performed with the GraphPad Prism 5 software (version 5.01, GraphPad Software, San Diego, CA). The significance of differences between the control and samples treated with various compounds was determined by one-way ANOVA followed by Tukey’s test*.* Differences were considered significant at *P* < 0.05.

## Results

### An agonist of H2R or H3R inhibits exploratory laparotomy-induced cognitive decline in rats

**Fig. 1:**
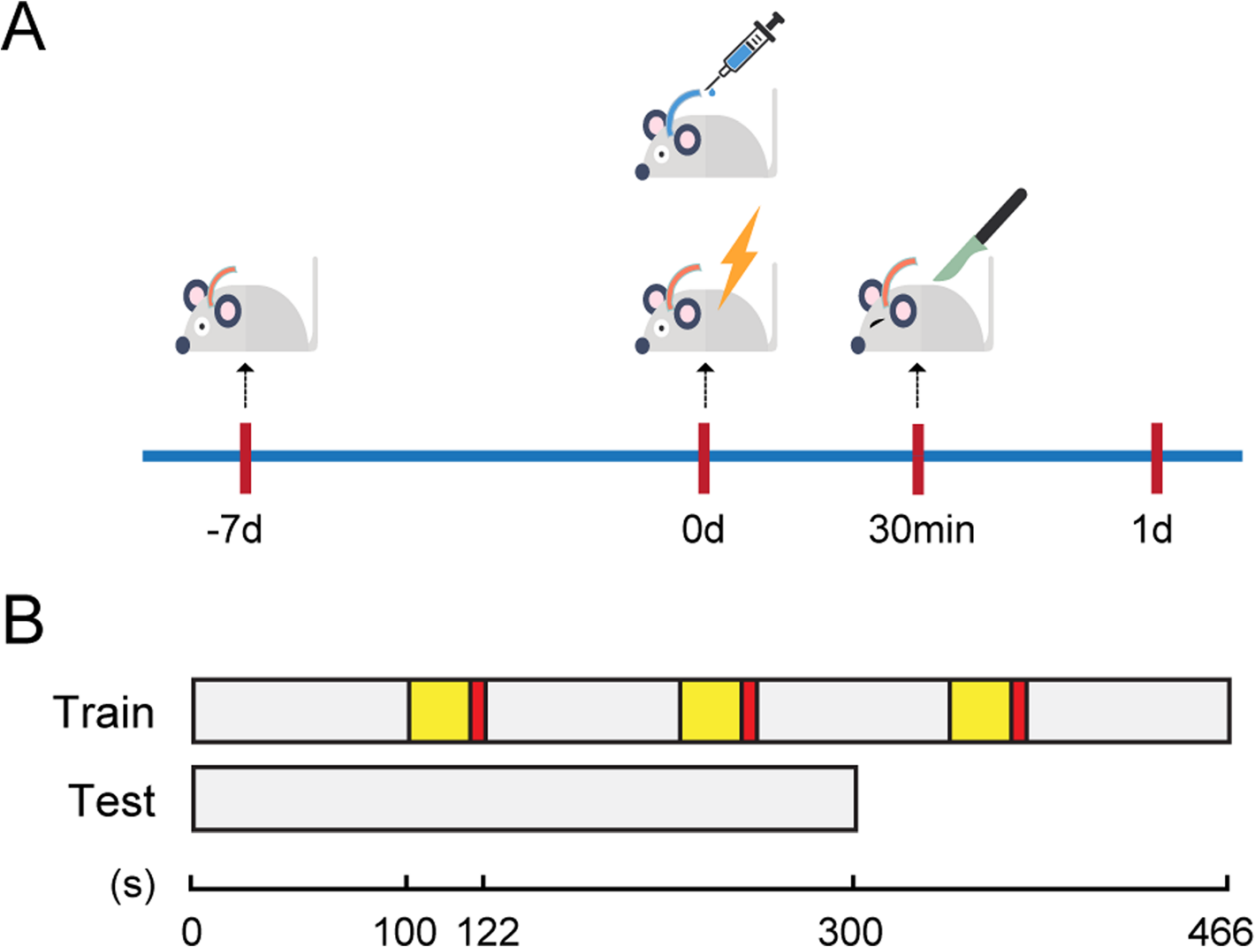
A schematic representation of the operation and administration timeline in rats. **a** Rats were anesthetized and inserted a guide cannula into the right lateral ventricle. After 7 days, the rats received trace fear conditioning (TFC) training, and then drugs were injected into the right lateral ventricle through the implanted guide cannula. Rats were given exploratory laparotomy 30 min later, and tests were carried out 1 day after laparotomy. **b** A schematic representation of the TFC procedure in rats. In the TFC training part, the rats first explored 100 s, and then experienced three cycles of stimulation: 20 s of sound stimulation (yellow), 2 s of electrical stimulation (red), and 100 s of interval (grey). In the TFC test part, freezing behaviour in the absence of the tone and shock was automatically scored over the course of 300 s

**Fig. 2:**
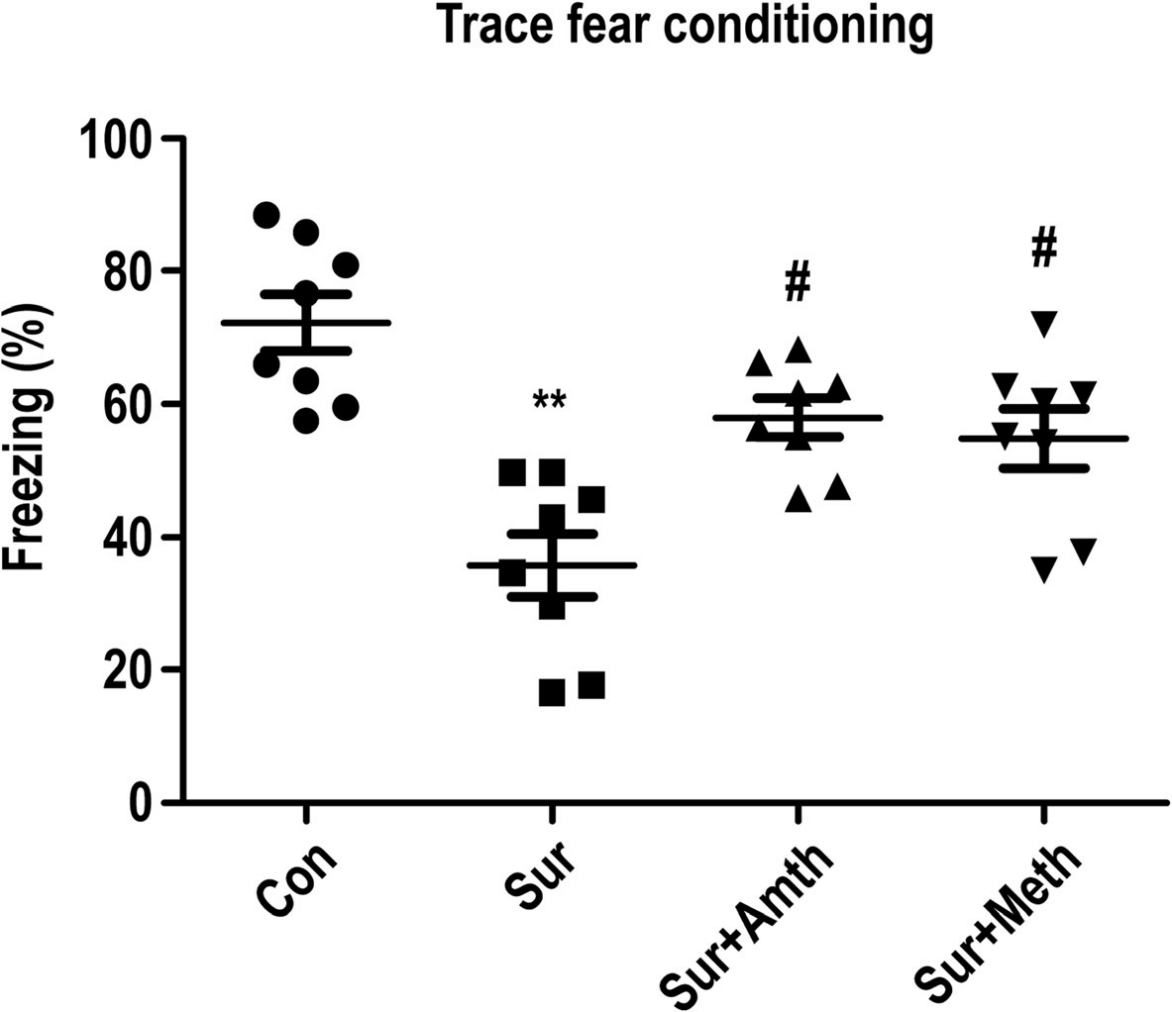
An agonist of H2R or H3R inhibits exploratory laparotomy-induced cognitive decline in rats. **P < 0.01 versus the control group. ^#^*P* < 0.05 versus the surgery group (*n* = 8). The data are presented as the mean ± SEM

In the in vivo experiment, the rats were administered an injection of saline (5 μl) or H2R/H3R agonists (10 mM, 5 μl) in the right lateral ventricle, followed by exploratory laparotomy 30 min later. Cognitive function was subsequently assessed using the trace fear conditioning test 1 day after exploratory laparotomy (Fig. 1). As shown in Fig. 2, the rats who were treated with an injection of saline and exploratory laparotomy exhibited a significant reduction in cognitive function, as indicated by a reduction in freezing behavior compared to those administered only saline. Notably, H2R/H3R agonist pretreatment ameliorated the decline in exploratory laparotomy-induced freezing behavior. Therefore, these results indicate that H2R and H3R agonists can ameliorate the adverse cognitive outcomes caused by exploratory laparotomy in rats.

### An agonist of H2R or H3R inhibits exploratory laparotomy-induced microglia activation and TLR4 expression in the rat hippocampus

**Fig. 3:**
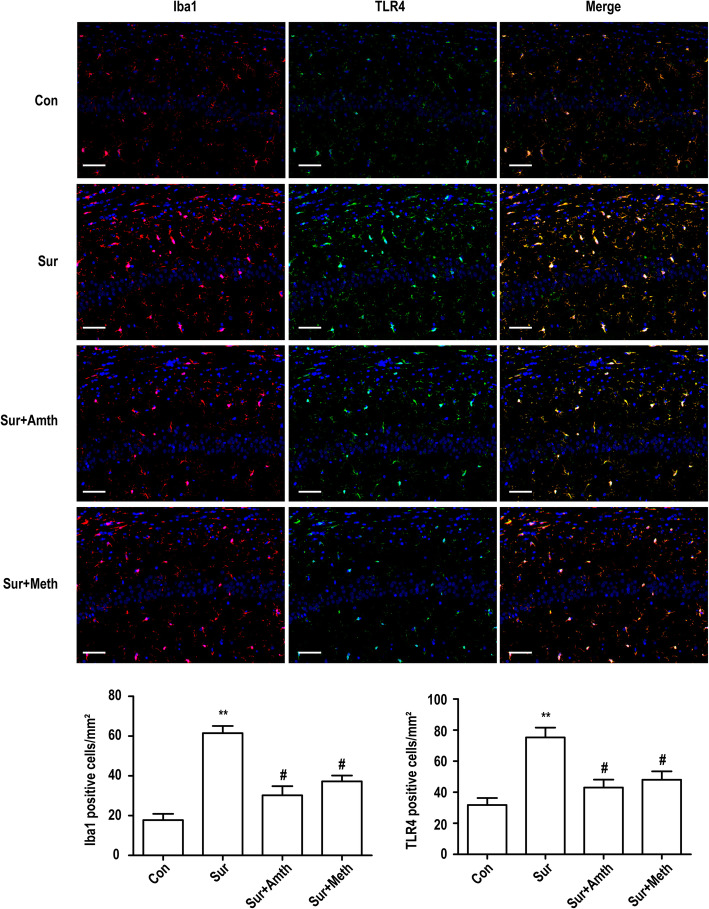
An agonist of H2R or H3R inhibits microglia activation and TLR4 expression in rat hippocampus. Immunofluorescent staining was used to detect Iba1 (red) and TLR4 (green) in hippocampal CA1 region. Blue staining represents DAPI. Scale bar = 50 μm. Histogram represents the quantification of Iba1 and TLR4 positive cells in the hippocampal CA1 area. **P < 0.01 versus the control group. ^#^*P* < 0.05 versus the surgery group (*n* = 4). The data are presented as the mean ± SEM

The effects of the H2R/H3R agonists on exploratory laparotomy-induced microglia activation were determined by immunostaining for Iba1, which is a marker of microglia. As shown in Fig. 3, exploratory laparotomy significantly upregulated the expression of Iba1 in the right hippocampus in rats. Simultaneously, the expression of TLR4 in the right hippocampus in rats also significantly increased after exploratory laparotomy, and most of the TLR4-positive cells exhibited high expression of microglia markers. Pretreatment with an H2R/H3R agonist suppressed exploratory laparotomy-induced TLR4 expression and microglia activation in the hippocampus.

### An agonist of H2R or H3R inhibits exploratory laparotomy-induced proinflammatory factor production in the rat hippocampus

**Fig. 4:**
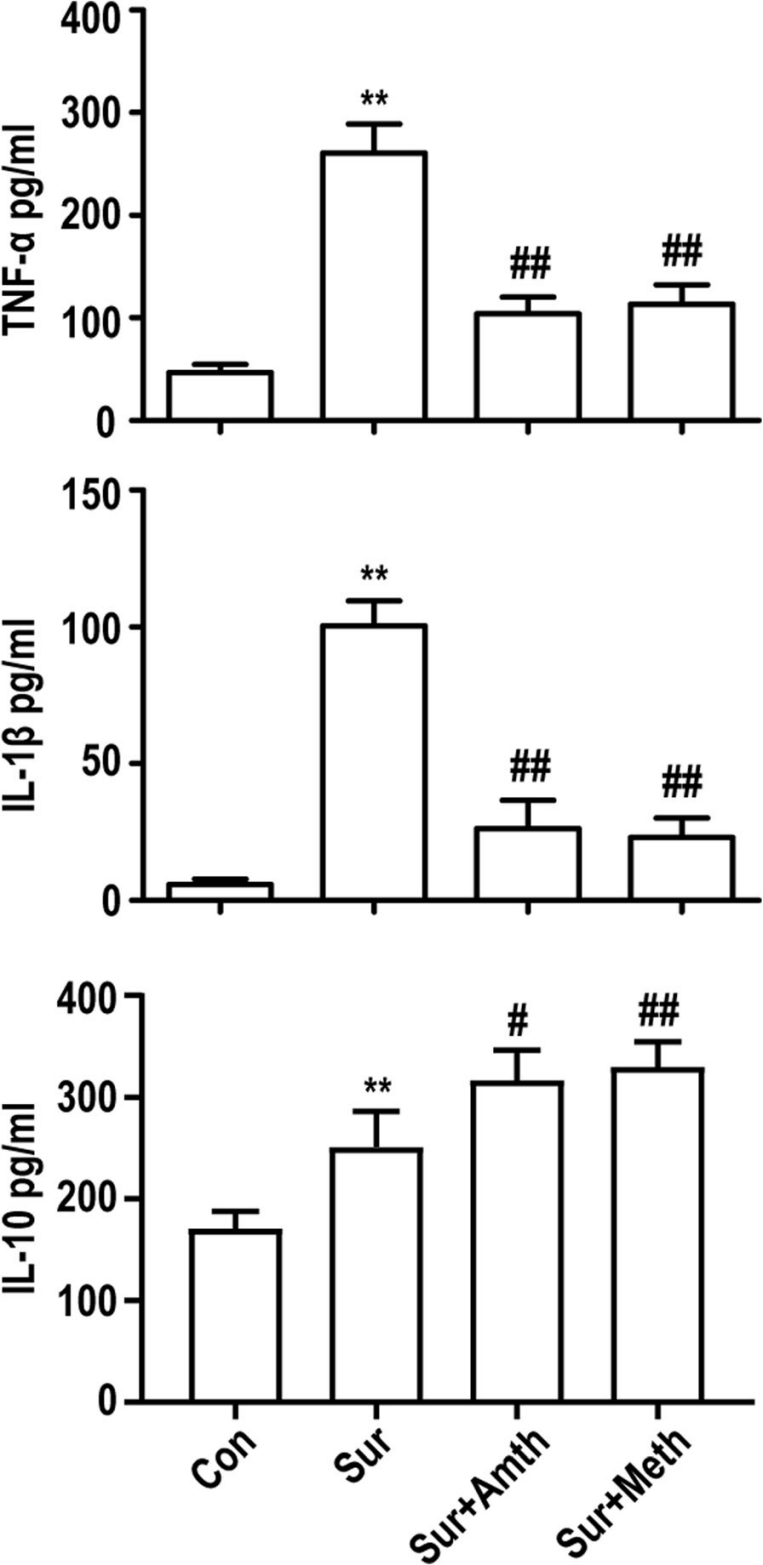
An agonist of H2R or H3R inhibits exploratory laparotomy-induced inflammatory factor production in rat hippocampus. The levels of the proinflammatory factors TNF-ɑ, IL-Iβm and IL-10 were detected by ELISA. **P < 0.01 versus the control group. ^#^*P* < 0.05, ^##^*P* < 0.01 versus the surgery group (*n* = 4). The data are presented as the mean ± SEM

Increasing evidence has demonstrated that excessive release of proinflammatory cytokines is involved in microglia-mediated neuroinflammation. The proinflammatory cytokines TNF-ɑ and IL-Iβ and the anti-inflammatory cytokine IL-10 were measured in the present study. We found that exploratory laparotomy significantly promoted TNF-ɑ, IL-Iβ, and IL-10 production in the ipsilateral hippocampus 24 h after the procedure. Injection of the H2R/H3R agonists 30 min before exploratory laparotomy partly inhibited the production of TNF-ɑ and IL-1β and increased the production of IL-10 in the ipsilateral hippocampus. These results indicated that the H2R/H3R agonists prevent exploratory laparotomy-induced proinflammatory cytokine release and increase anti-inflammatory cytokine release (Fig. 4).

### LPS-induced downregulation of H2R and H3R expression in microglia

**Fig. 5:**
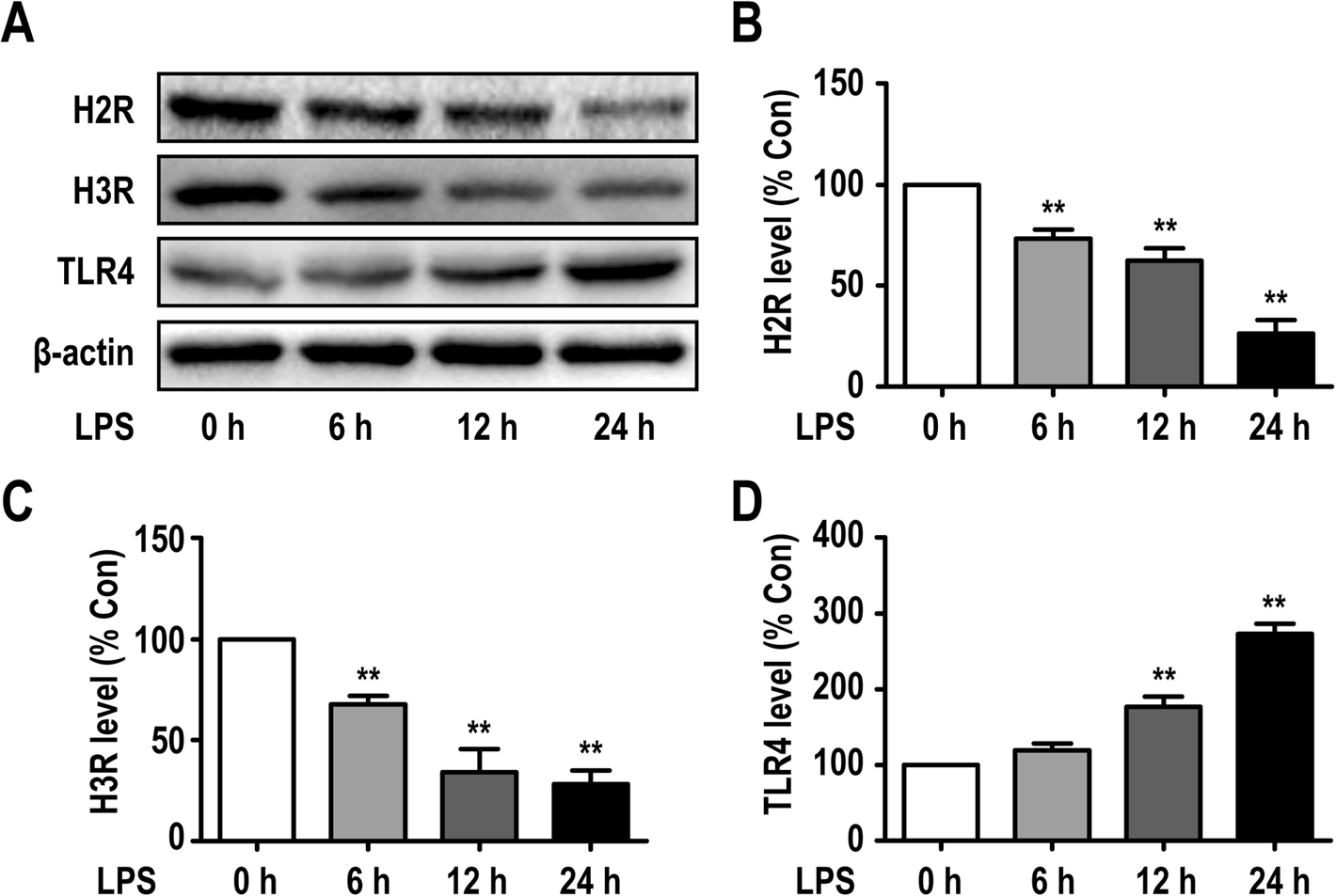
LPS induced downregulation of H2R and H3R expression in microglia. **a** The protein levels of H2R, H3R, and TLR4 were detected in primary cultured microglia by Western blotting using specific antibodies. **b–d** Expression of H2R, H3R, and TLR4 was quantified and normalized to β-actin levels. Each value was expressed relative to that in the control group (0 h), which was set to 100. **P < 0.01 versus the control group (*n* = 3). The data are presented as the mean ± SEM

We have previously shown that rat primary microglia express all four histamine receptors (H1R, H2R, H3R, and H4R); histamine at concentrations of 0.001 to 1 μg/ml selectively promoted the upregulation of H1R and H4R expression in a dose-dependent manner after a 24-h incubation period, but histamine had no effect on the expression of H2R or H3R [22]. In the present study, to determine whether LPS modulates the protein expression levels of histamine receptors in primary microglia, western blot analysis was used (Fig. 5a–c). The results showed that 10 ng/ml LPS promoted the downregulation of H2R and H3R expression in primary cultured microglia in a time-dependent manner. The downregulated expression of H2R and H3R was observed at 6 h and continued for at least 24 h.

In addition, we further showed that the expression levels of TLR4 on microglia were significantly upregulated by LPS in different time course studies; this upregulation of TLR4 was first observed at 12 h and lasted at least 24 h (Fig. 5a, d).

### An agonist of H2R or H3R inhibits LPS-induced microglia activation

**Fig. 6:**
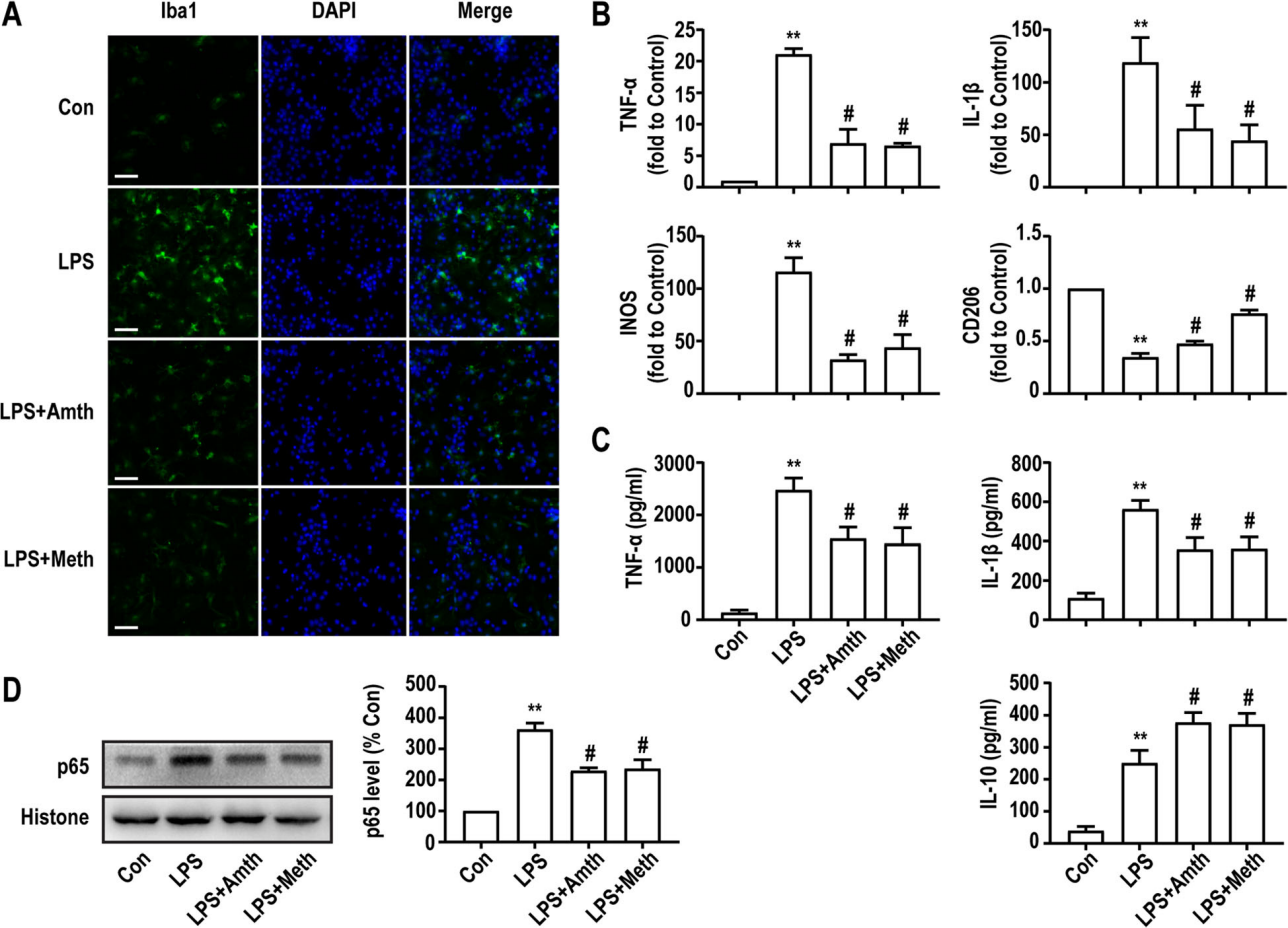
An agonist of H2R or H3R inhibits LPS-induced microglia activation and associated inflammatory response. **a** Primary microglia were stained with Iba1 antibody as indicated. Blue staining represents DAPI. Scale bar = 50 μm. **b** q-PCR analysis of the relative expression of TNF-α, IL-1β, INOS, and CD206 mRNA. Each value was expressed relative to that in the control group, which was set to 1. **c** Concentration of TNF-α, IL-1β, and IL-10 measured by ELISA. **d** The expression levels of NF-κb were detected in primary cultured microglia by Western blotting using specific antibodies. Expression of NF-κb was quantified and normalized to histone levels. Each value was expressed relative to that in the control group, which was set to 100. **P < 0.01 versus the control group. ^#^*P* < 0.05 versus the LPS group (*n* = 3). The data are presented as the mean ± SEM

To further confirm the protective effects of H2R/H3R receptors in vitro, primary microglia were labeled with Iba1 by immunofluorescence. Primary microglia were pretreated with the H2R/H3R agonists (10 μM) and then incubated with LPS (10 ng/ml) 30 min later. As shown in Fig. 6a, after incubation with LPS for 24 h, Iba1 expression in microglia was markedly upregulated compared with the expression observed in the control group. However, pretreatment with the H2R/H3R agonists markedly inhibited the effect of LPS. These results suggest that LPS induces the activation of microglia, which can be suppressed by H2R/H3R agonists.

### An agonist of H2R or H3R promotes microglia polarization to the M2 phenotype

Since microglia can polarize into two reactive states, which are classified as M1 and M2 phenotypes, and the balance between these phenotypes is necessary to maintain CNS homeostasis, we used different markers to assess the role of H2R/H3R in microglia phenotypic transformation. The PCR results showed that compared with those of the control group, the mRNA levels of M1 microglia markers (TNF-α, IL-1β, and INOS) were significantly increased, and expression of the M2 microglia marker (CD206) was significantly decreased after LPS challenge for 24 h. However, H2R/H3R agonists could suppress the effect of LPS, downregulating M1 marker expression and upregulating M2 microglia marker expression compared with those in the LPS group (Fig. 6b).

### An agonist of H2R or H3R inhibits LPS-induced TNF-α/IL-1β production and NF-κb expression

Microglia actively participate in neuroinflammation via the excessive secretion of inflammatory factors, and the levels of inflammatory mediators (TNF-α, IL-1β, and IL-10) were analyzed by ELISA. As shown in Fig. 6c, compared with the control group, the group treated with 10 ng/ml LPS for 24 h had enhanced expression of TNF-α, IL-1β, and IL-10. The H2R agonist amthamine (10 μM) and H3R agonist (R)-(−)-α-methylhistamine (10 μM) partially abolished LPS-induced TNF-α and IL-1β release and upregulated IL-10 release.

Furthermore, we investigated the expression level of NF-κb in microglia. As shown in Fig. 6d, LPS upregulated NF-κb expression, and this effect was inhibited by the H2R/H3R agonists. These results indicated that the H2R/H3R agonists could suppress LPS-induced proinflammatory factor release, increase anti-inflammatory factor release, and inhibit NF-κb expression in primary microglia.

### An agonist of H2R or H3R inhibits LPS-induced microglia migration

**Fig. 7:**
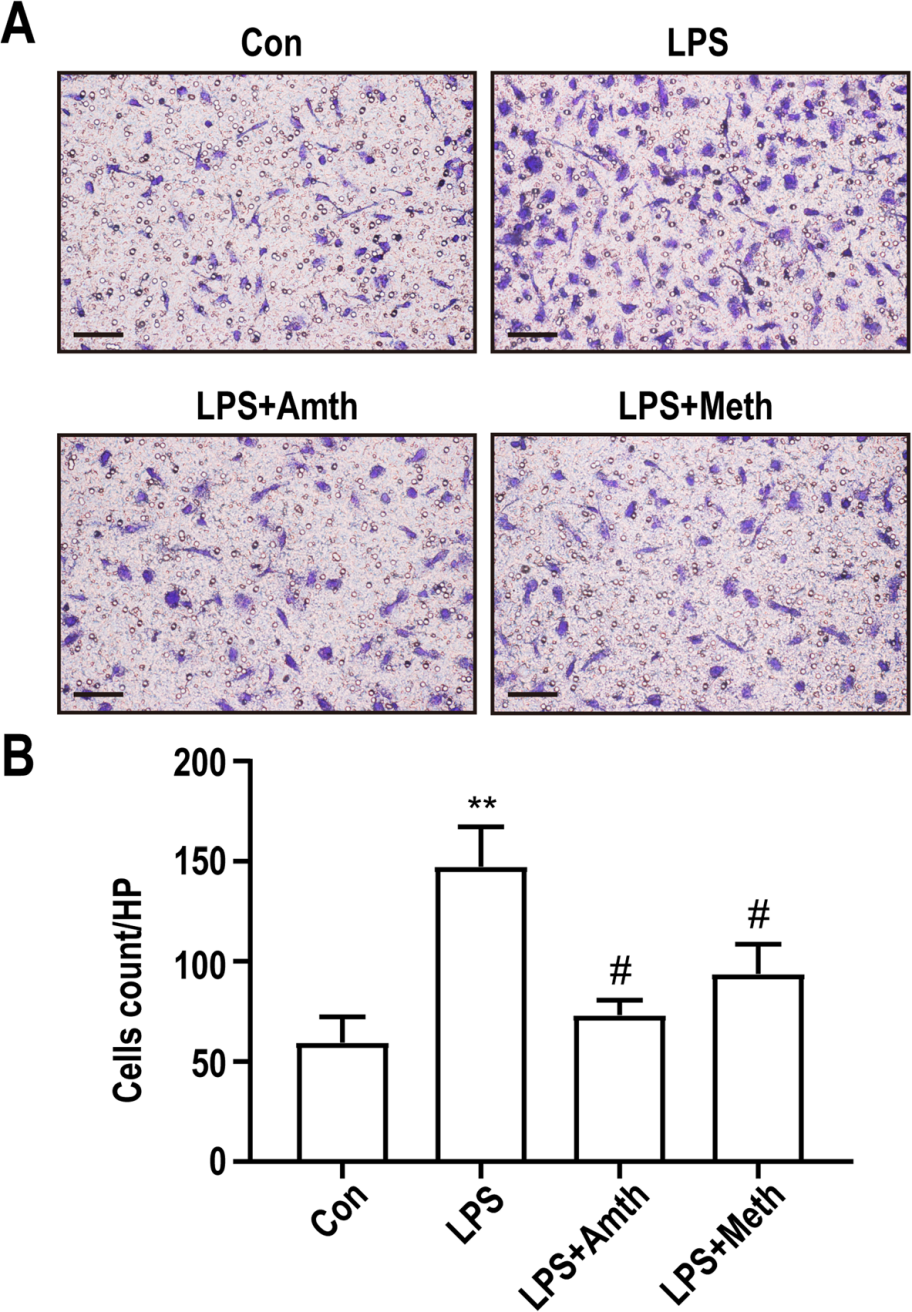
An agonist of H2R or H3R inhibits LPS-induced microglia migration. **a** The migration ability of primary microglia was tested by transwell assay. Scale bar = 20 μm. **b** Quantification of primary microglia transmitted to the lower surface of the membrane. **P < 0.01 versus the control group. ^#^*P* < 0.05 versus the LPS group (*n* = 3). The data are presented as the mean ± SEM

We used a transwell assay to analyze the migration abilities of primary microglia. As shown in Fig. 7, incubation with 10 ng/ml LPS for 24 h induced a significant increase in microglia migration compared with that in the control group. However, H2R/H3R agonists partially inhibited LPS-induced microglia migration.

### An agonist of H2R or H3R inhibits LPS-induced TLR4 expression by activating PI3K/AKT/FoxO1 signaling

**Fig. 8:**
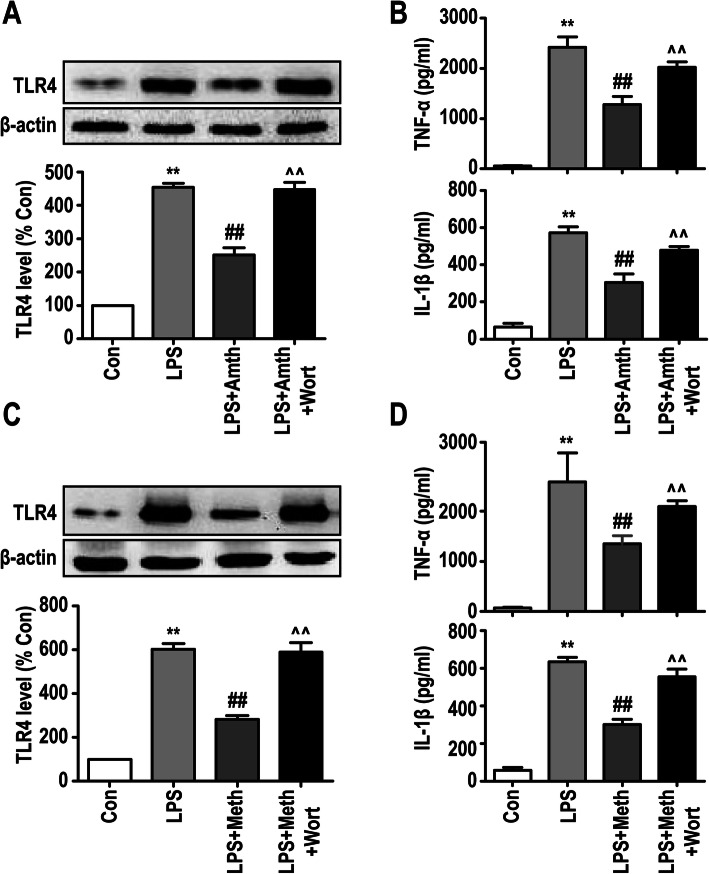
An agonist of H2R or H3R inhibits LPS-induced TLR4 expression by activating PI3K/AKT signaling. **a, c** The protein levels of TLR4 were detected by Western blotting using specific antibody in primary microglia, and expression of TLR4 was quantified and normalized to β-actin levels. Each value was expressed relative to that in the control group, which was set to 100. **b, d** Concentration of TNF-α and IL-1β measured by ELISA. **P < 0.01 versus the control group. ^##^*P* < 0.01 versus the LPS group. ^^^^*P* < 0.01 versus the LPS + Amth group. ^^^^*P* < 0.01 versus the LPS + Meth group (*n* = 3). The data are presented as the mean ± SEM

The in vivo experiments revealed that exploratory laparotomy promoted TLR4 expression and inflammatory factor release in the rat hippocampus, which could be suppressed by the H2R/H3R agonists. To further study the mechanism by which H2R and H3R affect microglia-induced inflammation, we first examined the effect of H2R and H3R on TLR4 expression in primary microglia. As shown in Fig. 8a, c, LPS upregulated the expression of TLR4 compared with that of the control group. However, H2R and H3R agonists partly inhibited the expression of TLR4. These results suggest that the H2R/H3R agonists can inhibit LPS-induced TLR4 pathway activation in primary microglia. In addition, we used wortmannin to inhibit the PI3K/AKT pathway, since it is the downstream pathway of TLR4. The results showed that 10 μM wortmannin abolished H2R/H3R agonist-mediated suppression of TLR4 expression in primary microglia, suggesting that the H2R/H3R agonists could inhibit TLR4 expression through the PI3K/AKT pathway. Furthermore, the production of inflammatory cytokines was examined by ELISA, and the results showed that the H2R/H3R agonists suppressed LPS-induced TNF-α and IL-1β release in primary microglia, which was inhibited by the PI3K/AKT inhibitor wortmannin (Fig. 8 b, d).

**Fig. 9:**
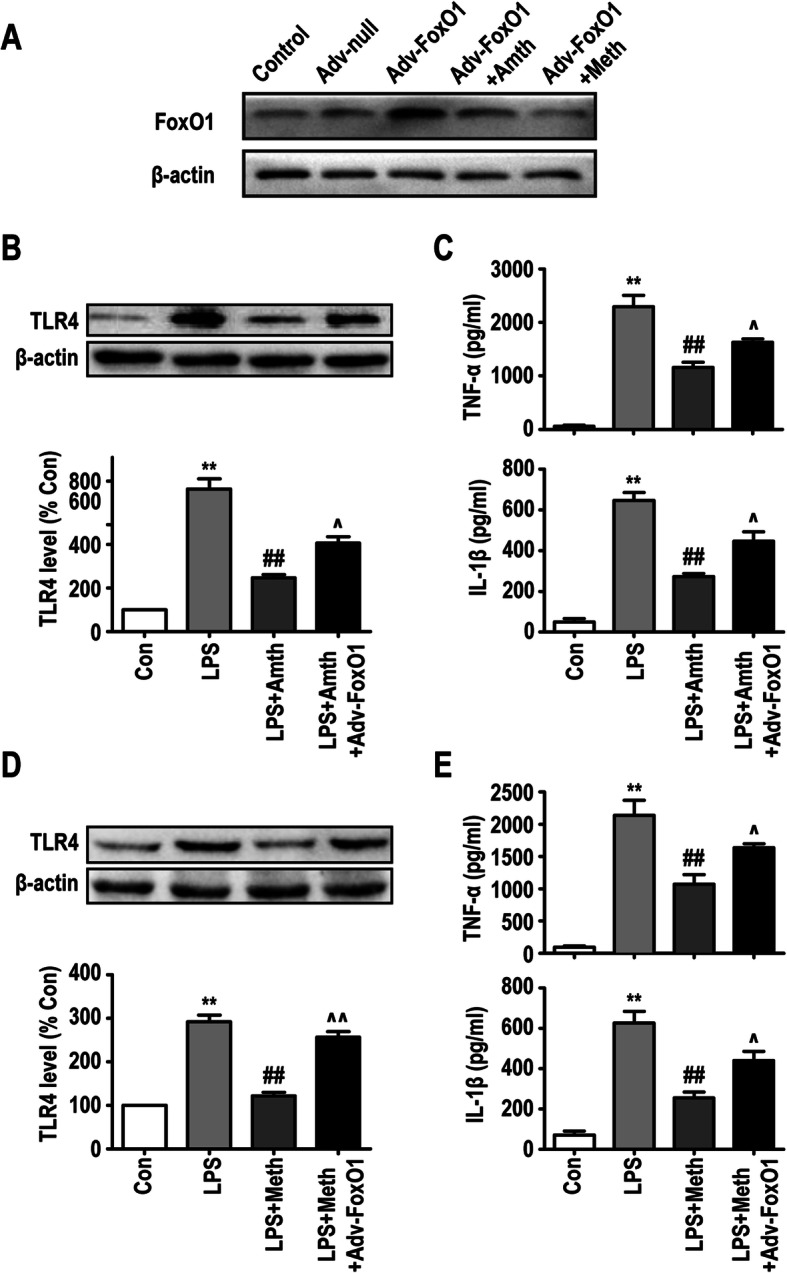
FoxO1 participates in H2R/H3R-mediated microglia TLR4 expression and inflammatory factor release. **a** The protein levels of FoxO1 were detected by Western blotting using specific antibody in primary microglia. **b, d** The protein levels of TLR4 were detected by Western blotting using specific antibody in primary microglia, and expression of TLR4 was quantified and normalized to β-actin levels. Each value was expressed relative to that in the control group, which was set to 100. **c, e** Concentration of TNF-α and IL-1β measured by ELISA. **P < 0.01 versus the control group. ^##^*P* < 0.01 versus the LPS group. ^^^*P* < 0.05 versus the LPS + Amth group. ^^^*P* < 0.05 and ^^^^*P* < 0.01 versus the LPS + Meth group (*n* = 3). The data are presented as the mean ± SEM

The transcriptional activity of FoxO1 can be inhibited by the phosphorylation of PI3K/AKT, leading to nuclear translocation of FoxO1 and inhibition of TLR4 gene expression. To verify whether FoxO1 was involved in the H2R/H3R-mediated regulation of TLR4 signaling, we used Adv-FoxO1 to overexpress FoxO1 in primary microglia. As shown in Fig. 9a, Adv-null did not alter the expression of FoxO1, while Adv-FoxO1 effectively upregulated the expression of FoxO1 in primary microglia. H2R/H3R agonists inhibited Adv-FoxO1-induced FoxO1 expression. In addition, Adv-FoxO1 partly inhibited the effect of the H2R/H3R agonists in reducing TLR4 expression during LPS challenge (Fig. 9b, d). The production of TNF-α and IL-1β was also examined by ELISA, and the result showed that Adv-FoxO1 suppressed the effect of the H2R/H3R agonists in inhibiting inflammatory cytokine release in primary microglia (Fig. 9c, e).

## Discussion

It is important to note that neuroinflammation has become a key feature in the development of PND-associated neurological complications, and microglia-induced inflammation has been implicated in triggering neuronal death and CNS disfunction [32–34]. Increasing evidence indicates that histamine modulates microglia-mediated neuroinflammation through activating histamine receptors [15]. However, we have not developed a deep understanding of the molecular mechanisms of the four histamine receptors in microglia-induced inflammation. In particular, the mechanism by which the H2 and H3 receptors affect microglia-mediated inflammation is still unclear. In our present study, we reported that microglia H2R and H3R play protective roles in inflammation in both in vivo and in vitro experiments, as demonstrated by the H2R and H3R agonists partly inhibiting the activation of microglia, reducing the release of TNF-α and IL-1β, and suppressing the expression of NF-κb. Furthermore, to the best of our knowledge, we discovered for the first time that the underlying mechanisms of H2R and H3R were associated with PI3K/AKT/FoxO1 intracellular signaling.

Several studies have demonstrated that microglia play a crucial role in neuroinflammation. Different proinflammatory stimuli, such as LPS, histamine, and IL-10, can activate microglia and induce them to transform into the M1 or M2 inflammatory phenotype, which indicates an inflammatory or anti-inflammatory state, respectively [10, 35]. Among these stimuli, histamine has attracted much attention in the context of microglia-mediated inflammation. In our previous studies, we demonstrated that histamine could be released by mast cells (MCs) and induce neuroinflammation by activating microglia both in vivo and in vitro [36, 37]. However, some other studies showed that histamine can inhibit the proinflammatory effect of microglia during LPS challenge [38, 39]. Based on these results, we hypothesized that the four histamine receptors in microglia have different pathophysiological features. Under various environmental stimuli, different histamine receptors could be specifically activated or inhibited, and the relatively highly expressed “dominant receptor” may determine the progression of inflammation.

Supporting our hypothesis, previous studies have reported different effects of the four histamine receptors. Sandra M. Rocha discovered that histamine-mediated activation of H1R-induced microglia activation and ultimately the death of susceptible dopaminergic neurons both in mice and primary murine microglia [40]. Norihito Hiraga suggested that the enhancement of central histaminergic activity suppressed the recruitment of inflammatory cells after ischemic events through the histamine H2 receptor [41]. In addition, the H3R agonist imetit was used to show the anti-inflammatory effect of H3R by suppressing the secretion of TNF-α and PGE2 in primary microglia, while the H4R antagonist J7777120 reduced the activation of microglia and decreased the plasma levels of IL-1β and TNF-α in a rat model [38]. Consistent with these results, we previously discovered that histamine at concentrations of 0.001 to 1 μg/ml selectively upregulated the expression of H1R and H4R in a dose-dependent manner and increased the release of TNF-α and IL-6 in primary microglia [22]. In the present study, we found that 10 ng/ml LPS downregulated the expression of H2R and H3R in a time-dependent manner. These results demonstrated that different stimuli could activate or inhibit different receptors and influence the direction of inflammatory progression.

In the present study, to examine the roles of H2R and H3R in microglia-related inflammation, rats were directly injected with H2R/H3R agonists in the right lateral ventricles and then subjected to exploratory laparotomy 30 min later. We discovered that the expression of Iba1 in the right hippocampus in rats was significantly upregulated 1 day after exploratory laparotomy, while pretreatment with the H2R/H3R agonists suppressed exploratory laparotomy-induced microglia activation in the hippocampus. In addition, pretreatment with the H2R/H3R agonists inhibited TNF-α and IL-1β expression but increased IL-10 expression in the hippocampus in rats that underwent exploratory laparotomy. These results could be supported by a mouse model with a disrupted H3 receptor (H3RKO), in which more severe disease and neuroinflammation developed compared with those of wild-type animals [42]. Current studies indicate that neuroinflammation plays a vital role in the pathogenesis of several disorders and can induce cognitive decline [43–45]. Thus, we further examined the cognitive function of the rats and discovered that rats that underwent exploratory laparotomy experienced serious cognitive declines in trace fear conditioning, while those that were pretreated with the H2R/H3R agonists in the lateral ventricles did not show obvious cognitive differences. These results indicated that H2R/H3R may play a protective role in microglia-related CNS inflammation and inflammation-related neurological disease. However, peripheral surgery could activate not only microglia but also astrocytes and MCs. Similarly, the H2R/H3R agonists may affect several resident CNS immune cell types. Therefore, we further investigated the role and cellular mechanism of H2R/H3R in primary microglia-mediated inflammation.

It has been reported that primary microglia can be activated by 10 ng/ml LPS [46, 47]. In our in vitro experiments, primary microglia were pretreated with 10 μM H2R/H3R agonists 30 min before exposure to 10 ng/ml LPS. We found that LPS could activate primary microglia, induce the transformation of cells to the inflammatory M1 phenotype, increase the release of TNF-ɑ, IL-1β and IL-10, and upregulate the expression of NF-κb and TLR4. However, pretreatment with H2R/H3R agonists could suppress the effects of LPS, which was consistent with our in vivo results. In addition, pretreatment with 10 μM H2R/H3R agonists also restrained the migration of primary microglia induced by 10 ng/ml LPS. However, the effects of the H2R/H3R agonists only partly inhibited but did not fully reverse the effect of LPS.

TLR4, a member of the TLR family, is traditionally accepted to be a crucial mediator in microglia-induced neuroinflammation, which results in neurodegeneration both in vivo and in vitro [48, 49]. In this study, we demonstrated that TLR4 actively participated in the anti-inflammatory effects of H2R/H3R as follows: (i) the expression of TLR4 in the rat hippocampus significantly improved 1 day after exploratory laparotomy, while pretreatment with the H2R/H3R agonists inhibited the expression of TLR4, and (ii) the expression of TLR4 in primary microglia was upregulated by LPS, but pretreatment with the H2R/H3R agonists reduced the expression of TLR4 during LPS challenge. Notably, in our in vivo experiments, the increase in the TLR4-positive cell population in the hippocampus was accompanied by an increase in Iba1-positive cells after exploratory laparotomy. The number of TLR4-positive cells was greater than that of Iba1-positive cells, but most TLR4-positive cells also exhibited high expression of Iba1. These findings strongly indicate that exploratory laparotomy can upregulate TLR4 expression in a variety of resident immune cells in the rat hippocampus, with microglia accounting for a large proportion.

**Fig. 10:**
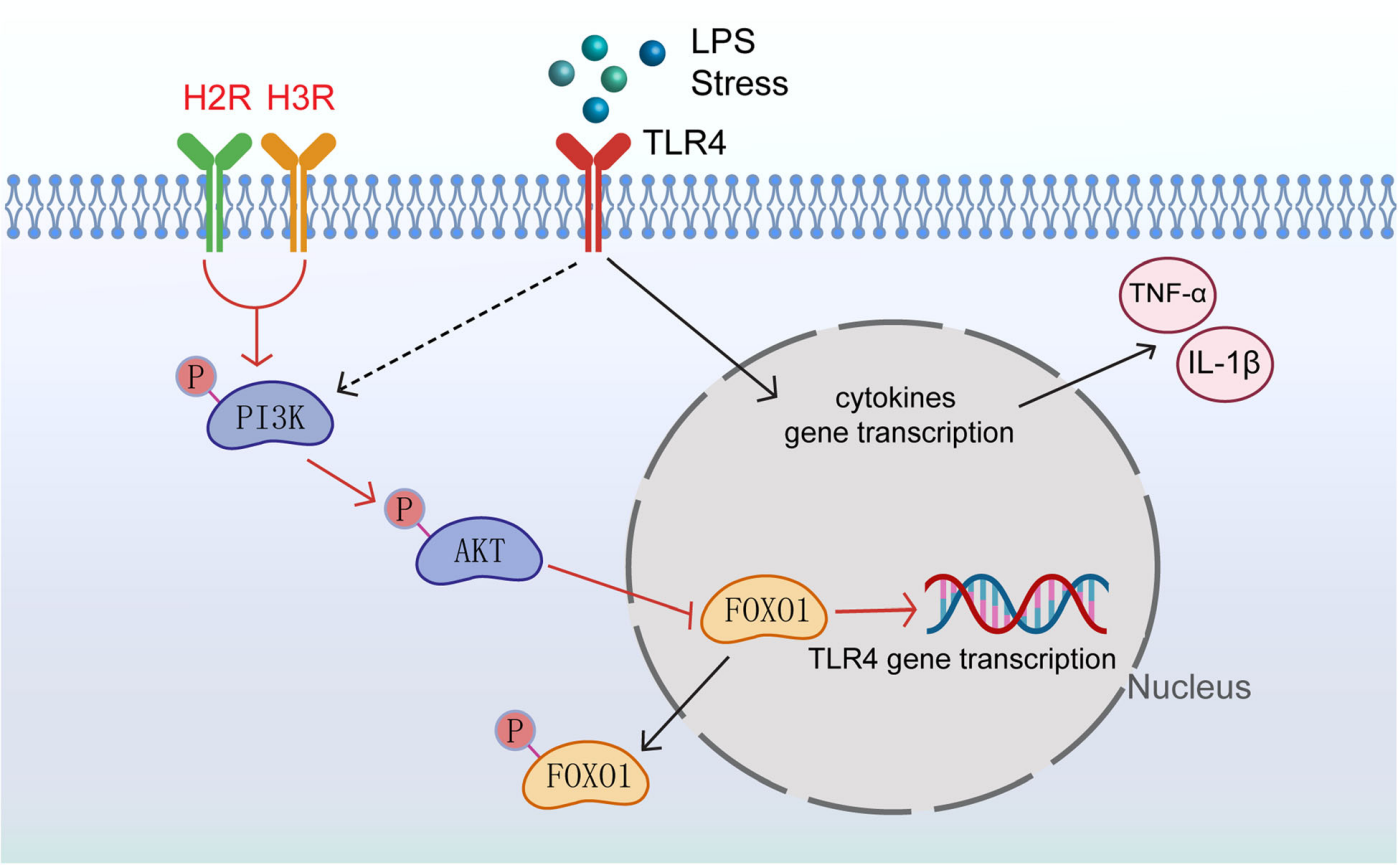
A schematic illustration of the proposed mechanism for H2R/H3R to alleviate microglia-induced neuroinflammation. LPS or stress activates TLR4 and induces microglia to release inflammatory cytokines. PI3K/AKT/FoxO1 axis, as the downstream of TLR4, provides a self-limiting mechanism by which microglia avoid inappropriate long-term overactivation. H2R/H3R can activate PI3K/AKT/FoxO1 signaling, trigger the phosphorylation and nuclear exclusion of FoxO1, and inhibit microglia TLR4 expression and TLR4-mediated inflammatory cytokine release

In addition to the traditionally recognized proinflammatory effects of the TLR4 signaling pathway, there is a self-limiting pathway downstream of TLR4 that can inhibit TLR4-induced inflammation. FoxO1 is a downstream gene of TLR4, and the transcriptional activity of FoxO1 is inhibited through phosphorylation by PI3K/Akt, resulting in nuclear exclusion of FoxO1 and target gene downregulation. However, as a transcription factor, FoxO1 inactivation can consequently downregulate TLR4 gene expression, which is called “a self-limiting mechanism” in the TLR4 pathway [28, 29, 31]. It was reported that baicalin can significantly increase the phosphorylation of PI3K/AKT/FoxO1 and lead to the downregulation of TLR4 expression in primary microglia [50]. Therefore, we hypothesized that the underlying mechanisms of H2R/H3R are related to the PI3K/AKT/FoxO1 signaling pathway. Our results showed that the H2R/H3R agonists downregulated the expression of TLR4 during LPS challenge, which could be abolished by the PI3K/AKT inhibitor wortmannin or FoxO1 overexpression by Adv-FoxO1 in primary microglia. In addition, the PI3K/AKT inhibitor wortmannin or Adv-FoxO1 also abolished the effect of the H2R/H3R agonists on the release of TNF-α and IL-1β in primary microglia. Therefore, we demonstrated that H2R/H3R might regulate the expression of TLR4 by activating the PI3K/AKT/FoxO1 pathway (Fig. 10).

Histamine receptors (H1R, H2R, H3R, and H4R) belong to the superfamily of G-protein coupled receptors (GPCRs). H2R is a preferential Gs/adenylyl cyclase-coupled receptor, and H3R is a Gi/Go-coupled receptor [51]. It has been reported that GPCRs can activate the PI3K/AKT signaling pathway. For example, orexin receptor (OXR) activation promotes the upregulation of a Gs-cAMP-cAMP-responsive element signaling pathway and plays a key role in the PI3K/AKT signaling pathway [52, 53]. The succinate receptor GPR91, a Gi-coupled receptor, can increase intracellular calcium concentrations through PLCb and upregulate the levels of p-Akt/t-Akt in cardiomyocytes [54, 55]. However, the specific mechanism of GPCR-mediated PI3K/AKT signaling still needs further study.

Activation of Gs protein leads to adenylate cyclase (AC) activity, followed by formation of the second messenger adenosine 3′,5′-cyclic monophosphate (cAMP) and PKA activity [56]. In addition, several studies reported that PKA could activate the PI3K/Akt pathway [57, 58]. Therefore, it is possible that H2R may activate cAMP-PKA and result in PI3K/Akt pathway activation. Accumulating evidence has also shown that GsPCRs can activate AKT through transactivation of TrkB [59, 60]. For example, the adenosine 2A (A2A) receptor induces new synthesis of an immature TrkB isoform and signaling by the newly formed TrkB increases phosphorylation and activation of protein kinase B (pAkt) [61].

When activated, GPCRs catalyze the replacement of GDP by GTP bound to the α subunit and induce the dissociation of α-GTP from βγ dimers. βγ dimers were found to play a critical role in signa. It has been found that membrane-bound forms of βγ subunits of heterotrimeric G proteins can potently stimulate Akt activity, and stimulation of PI3K upon H3R activation likely depends on Gβγ-subunits from Gi/o proteins, which are known to activate PI3K [62, 63].

In this study, we examined HRs and initially discovered the role and mechanism of microglia HRs in inflammation. It is worth noting that the H2R agonist amthamine also has a weak inhibitory effect on H3R. In future studies, we will use appropriate viruses or transgenic mice to specifically knock out microglia histamine receptors to further understand their roles in central inflammation and rat behaviors.

## Conclusions

To the best of our knowledge, this study confirms that H2R and H3R in microglia suppress microglia activation and proinflammatory responses. We demonstrate for the first time that the possible intracellular signaling mechanism of H2R/H3R-mediated microglia activation and neuroinflammation is the PI3K/AKT/FoxO1 pathway. These results provide a novel mechanism by which H2R and H3R might play protective roles in microglia-induced neuroinflammation associated with PND, which could constitute new therapeutic targets for CNS immune inflammation-related diseases.

## Data Availability

The datasets supporting the conclusions of this article are included within the article. All materials used in this manuscript will be made available to researchers and are subject to confidentiality.

## Abbreviations

H2R: Histamine 2 receptor
H3R: Histamine 3 receptor
amthamine: Amthamine dihydrobromide
(R)-(−)-α-methylhistamine: (R)-(−)-α-methylhistamine dihydrobromide
LPS: Lipopolysaccharide
CNS: Central nervous system
PND: Perioperative neurocognitive disorders
TLR4: Toll-like receptor 4
PI3K: Phosphatidylinositol 3-kinase
AKT: Protein kinase B
FoxO1: Forkhead box O1
TNF-α: Tumor necrosis factor-α
IL-1β: Interleukin-1β
IL-10: Interleukin-10
Iba1: Ionized calcium-binding adapter molecule 1
MCs: Mast cells
ELISA: Enzyme-linked immunosorbent assay
PBS: Phosphate-buffered saline
